# Sublethal salinity stress contributes to habitat limitation in an endangered estuarine fish

**DOI:** 10.1111/eva.12385

**Published:** 2016-06-08

**Authors:** Lisa M. Komoroske, Ken M. Jeffries, Richard E. Connon, Jason Dexter, Matthias Hasenbein, Christine Verhille, Nann A. Fangue

**Affiliations:** ^1^ Department of Wildlife, Fish & Conservation BiologyUniversity of California at DavisDavisCAUSA; ^2^ Department of Anatomy, Physiology & Cell BiologySchool of Veterinary MedicineUniversity of California at DavisDavisCAUSA; ^3^ National Research Council under contract to Southwest Fisheries Science Center, National Marine Fisheries ServiceNational Oceanic and Atmospheric AdministrationLa JollaCAUSA

**Keywords:** anadromous fish, climate change, delta smelt, environmental stress, *Hypomesus transpacificus*, osmoregulation, transcriptome

## Abstract

As global change alters multiple environmental conditions, predicting species’ responses can be challenging without understanding how each environmental factor influences organismal performance. Approaches quantifying mechanistic relationships can greatly complement correlative field data, strengthening our abilities to forecast global change impacts. Substantial salinity increases are projected in the San Francisco Estuary, California, due to anthropogenic water diversion and climatic changes, where the critically endangered delta smelt (*Hypomesus transpacificus*) largely occurs in a low‐salinity zone (LSZ), despite their ability to tolerate a much broader salinity range. In this study, we combined molecular and organismal measures to quantify the physiological mechanisms and sublethal responses involved in coping with salinity changes. Delta smelt utilize a suite of conserved molecular mechanisms to rapidly adjust their osmoregulatory physiology in response to salinity changes in estuarine environments. However, these responses can be energetically expensive, and delta smelt body condition was reduced at high salinities. Thus, acclimating to salinities outside the LSZ could impose energetic costs that constrain delta smelt's ability to exploit these habitats. By integrating data across biological levels, we provide key insight into the mechanistic relationships contributing to phenotypic plasticity and distribution limitations and advance the understanding of the molecular osmoregulatory responses in nonmodel estuarine fishes.

## Introduction

Current and forecasted shifts in environmental conditions due to human activities are changing the sources, strengths and directions of selective pressures for organisms globally (Rice and Emery 2003). Species responses can vary greatly, including *in situ* adaptation and acclimatization (Palumbi et al. 2014), extirpation or extinction (Parmesan and Yohe 2003), and range and phenology shifts (Menzel et al. 2006; Pinsky et al. 2013). This myriad of responses is due in part to the fact that the conditions under which a species persists (*sensu* Hutchinson 1957) and exhibits optimal performance (Pörtner and Farrell 2008; Kassahn et al. 2009) may be strongly dominated by a single parameter, or a combination of environmental and ecological factors (e.g., physiological tolerances, predation pressure, resource availability; Brown 1984; Guisan and Thuiller 2005; Helmuth et al. 2005). Thus, as anthropogenically driven global change concurrently alters multiple environmental and ecological conditions, predicting species’ responses can be challenging without mechanistic understanding of how individual factors and their interactions affect species of interest. Integrative approaches that evaluate mechanistic responses can greatly complement correlative field data to quantify these relationships, strengthening our abilities to forecast species and ecological effects of global change.

In aquatic ecosystems, the effects of salinity in the context of global change have received less attention relative to other environmental factors such as temperature or carbonate chemistry. This has been in part due to inconsistency in the magnitude, direction, and variability of projected salinity changes due to both regional and global factors (IPCC 2013). While melting polar ice caps are decreasing mean salinities in the pelagic ocean (van Wijk and Rintoul 2014), sea‐level rise and drought conditions can raise salinities in coastal areas via flooding and seawater invasion of aquifers coupled with reduced freshwater inputs. Additionally, the increasing frequency and severity of extreme events (e.g., tsunamis or hurricanes) and storminess (Bromirski et al. 2003; Min et al. 2011) have the potential to drive large‐scale, rapid salinity fluctuations in estuarine habitats (Najjar et al. 2009, 2010; Cloern et al. 2011). To add further complexity, many of these drivers co‐occur with other anthropogenic activities that affect salinity. For example, in the San Francisco Estuary ecosystem (SFE; California, USA), in addition to strong natural tidal influences, salinity gradients are affected by both direct (e.g., freshwater diversion and flow regulation; Lund et al. 2010) and indirect anthropogenic activities (e.g., climate change induced heightened saltwater intrusion from sea‐level rise and reduced snowpack leading to diminished freshwater flows; Cloern et al. 2011; Cloern and Jassby 2012). Despite these complexities, salinity is a key abiotic factor limiting aquatic organisms (Nicol 1967), and therefore, it is critical to examine species’ responses to salinity changes to understand biological impacts under different global change scenarios.

Salinity poses challenges for aquatic organisms in large part because intra‐ and intercellular solute and water balance can strongly impact biochemical processes and physiological functions (Hochachka and Somero 2002). Unlike many invertebrates and some primitive fishes that conform to external salinities, teleost fishes osmoregulate to maintain an internal osmolality of approximately 300 mosmol kg^−1^ (Kültz 2015). This equates to isosomotic at roughly 9 ppt and strongly contrasts with external conditions in freshwater (<0.5 ppt) and marine (~33–40 ppt) environments (IAL and IUBS 1958). Sustaining this relatively constant internal state of ionic and osmotic equilibrium facilitates proper cell function, normal biological processes, and overall homeostasis, but requires adaptations to counteract passive salt loss and water gain in freshwater habitats or the converse in marine environments (Evans 2008a,b; Bradley 2009). For example, gills play a major role in transporting monovalent ions into or out of the body via mitochondrial rich cells (MRCs) and associated transport enzymes (e.g., Na^+^/K^+^‐ATPases), cotransporters (e.g., Na^+^/K^+^/Cl^−^ cotransporters, aquaporins), and other cellular structures (McCormick et al. 2003; Evans et al. 2005; Marshall and Grosell 2005; Hwang and Lee 2007).

**Figure 1:**
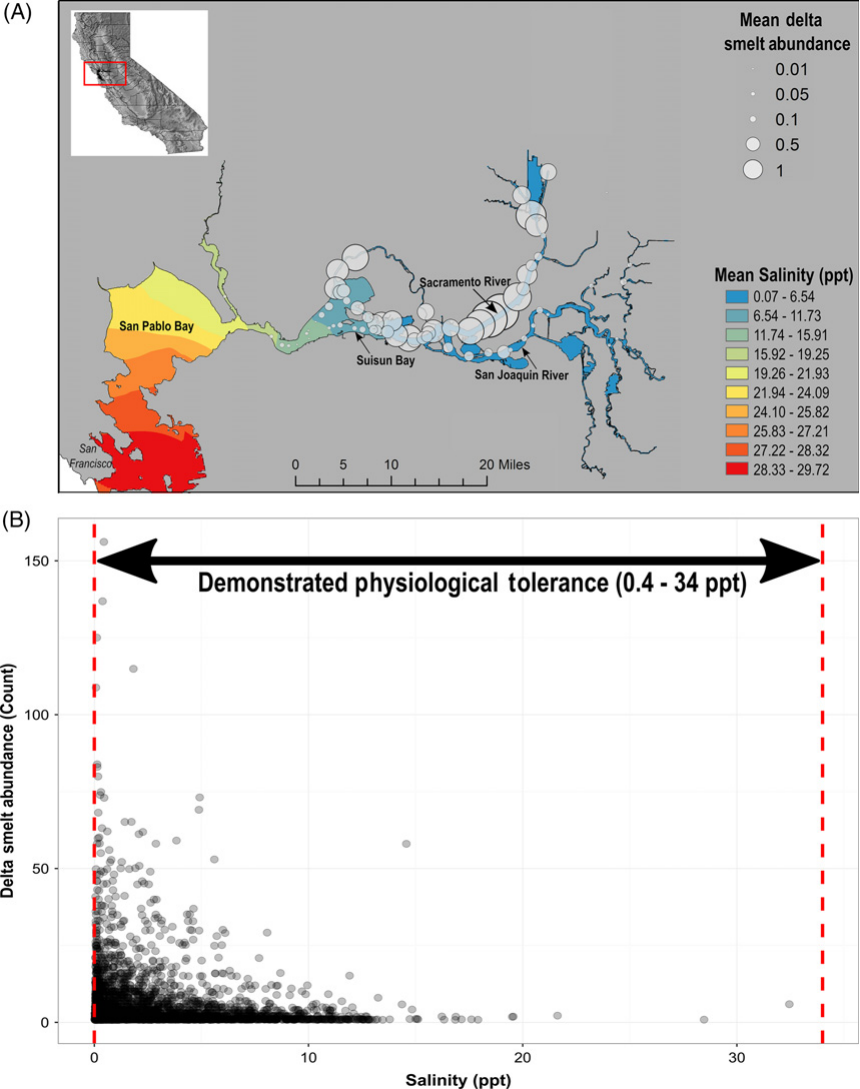
(A) Map of salinity seascape and adult delta smelt abundance in San Francisco Estuary; salinity interpolation based on mean salinity at each sampling station, and station symbols weighted to mean delta smelt abundance utilizing Fall Midwater Trawl Survey (FMWT) data 2000–2015. (B) Distribution of adult delta smelt in relation to environmental salinity during FMWT surveys 1967–2015, overlaid with their demonstrated physiological tolerance range from Komoroske et al. (2014).

Given the starkly opposing requirements for hyper‐ versus hyposmoregulation, it is perhaps unsurprising that the majority of fishes possess physiological adaptations to effectively osmoregulate in either static freshwater or saltwater conditions, but have limited capacity to tolerate salinity changes (termed stenohaline). However, some species, such as estuarine fishes that have evolved in dynamic ecosystems where salinity and other abiotic parameters greatly fluctuate, exhibit abilities to cope with large environmental salinity changes (termed euryhalinity; Marshall 2013; McCormick et al. 2013; Schultz and McCormick 2013). These organisms may utilize osmosensors to signal and induce large‐scale molecular cascades and cellular remodeling to adjust or even completely switch physiological strategies from hyper‐ to hyposmoregulation to restore homeostasis under widely fluctuating environmental salinities (Sardella et al. 2004; Fiol and Kültz 2007; Evans and Somero 2008; Evans 2010; Whitehead et al. 2011; Kültz 2013). Although such capabilities might, on the surface, suggest that salinity regime shifts due to global change will not negatively impact euryhaline fishes already adapted to dynamic salinity conditions, activation of the requisite osmotic compensatory responses can exert high sublethal costs (Kidder et al. 2006; Whitehead et al. 2012). Cellular remodeling associated with salinity transitions in euryhaline teleosts can include large‐scale protein synthesis, changes in activities of key enzymes such as Na^+^/K^+^‐ATPases, and growth or elimination of specialized cells (e.g., pavement cells, ionocytes, and seawater‐ or freshwater‐type MRCs; Evans et al. 2005; Marshall 2013). These alterations are energetically expensive and in turn can affect metabolic rates and energy balance, which play key roles in organisms’ survival, function, stress adaptation, and tolerance (Calow and Forbes 1998; Sokolova et al. 2012). Such osmoregulatory processes have been estimated to consume 20–68% of total energy costs in some teleosts (Morgan and Iwama 1991; Boeuf and Payan 2001), leading to substantial selective pressure to physiologically and behaviorally optimize osmoregulatory energetic expenditure (Calow 1989). Thus, optimal performance can occur at relatively narrow ranges within a larger physiological tolerance window, and fish may behaviorally avoid salinities outside this optimum range (Edeline et al. 2005; Dowd et al. 2010). Such mechanisms could contribute to observed patterns *in situ* in which a species’ occurrence is strongly bound within a small fraction of their tolerance range [defined here as physiologically euryhaline, but ecologically stenohaline, *sensu* Hutchinson's fundamental versus realized niche (1957); Fig. 1]. However, the physiological mechanisms and sublethal costs of such osmoregulatory responses from a molecular perspective are largely unresolved for fish that exhibit these patterns. Other estuarine, euryhaline fishes have been shown to use cytokine‐ and kinase‐signaling pathways to trigger complex transcriptional adjustments in response to changing salinities, including aquaporins to regulate cell volume, polyamine synthesis to stabilize protein interactions, ATPases to transport ions, claudins that may regulate paracellular permeability and ion selectivity, and a diversity of genes involved in energy metabolism and oxidative phosphorylation (Scott et al. 2005, 2008; Evans and Somero 2008; Whitehead et al. 2011; Marshall 2013). However, divergent salinity responses can exist even among populations (Scott and Schulte 2005; Whitehead et al. 2012), and most well‐studied species generally utilize habitats *in situ* across broad salinity ranges (i.e., are both physiologically and ecologically euryhaline; Marshall 2013). Additionally, there is strong evidence that euryhalinity as a phenotypic trait has evolved independently multiple times in different teleost lineages, and many proteins involved in euryhalinity actually exist in stenohaline fish but serve other biological functions (Schultz and McCormick 2013; Kültz 2015). Thus, it is unknown if the same molecular responses are conserved, muted, or absent in fish that can tolerate high salinities but do not exploit such habitats.

Understanding the biological consequences of current and projected salinity increases in highly managed systems like the SFE is particularly important because alternate management actions can strongly influence abiotic conditions and affect biodiversity and community structure. Human‐driven landscape‐scale modifications in the SFE have already reduced habitat diversity and led to major declines of once numerous native species (Sommer et al. 2007; Moyle et al. 2010), exemplified by precipitous declines of multiple pelagic fish populations since the early 2000s (referred to as the pelagic organism decline, POD; Feyrer et al. 2007; Sommer et al. 2007). One of the POD species is the delta smelt, *Hypomesus transpacificus*, an endangered pelagic species endemic to the SFE (Bennett 2005; CDFW 2014). The semi‐anadromous life history of delta smelt is composed of a largely annual life cycle in which larval fish develop in freshwater habitats until migrating downstream as juveniles toward the low‐salinity zone (LSZ; 1–6 ppt) where they typically rear until migrating back into freshwater as adults to spawn (Bennett 2005; Moyle et al. 2010). Thus, the earliest (eggs, larval phases) and latest (spawning adults) life stages experience freshwater conditions, while juvenile and prespawning adults largely experience low‐salinity conditions (i.e., hyposmotic). These latter stages behaviorly adjust their location according to the geographical position of the LSZ as it shifts in space and time due to fluctuations in freshwater flows (naturally and due to anthropogenic water diversion) and tidal forcing. Correlations of delta smelt abundance *in situ* with the LSZ (Bennett 2005; Feyrer et al. 2007) are so consistent that salinity conditions and isohaline position have been integrated into suitable habitat indicator indices (Jassby et al. 1995; Feyrer et al. 2011; Fig. 1). Yet the underlying mechanisms constraining delta smelt to the LSZ are not fully understood. They have been occasionally observed in waters up to 18 ppt (Bennett 2005) and can physiologically tolerate higher salinities in the laboratory (Swanson et al. 2000; Komoroske et al. 2014), but 92% of fish occurrence *in situ* is at or below 6 ppt (Fig. 1B; CDFW 2014). This is in contrast to other euryhaline species such as killifish that effectively tolerate and exploit fresh, brackish, and seawater habitats (Whitehead et al. 2012). Delta smelt may be limited to low‐salinity waters via sublethal costs of osmotic compensatory responses and subsequent reduced performance at higher salinities (Hasenbein et al. 2013), biotic interactions (e.g., food resources or predation pressure), or a combination of these factors. However, covariation of salinity gradients with other abiotic and ecological conditions *in situ* has made it particularly challenging to tease apart the effects of these factors, which may not change in concert under future climate change scenarios.

Cloern et al. (2011) forecasted mean salinity increases of 2.2–4.5 ppt in the SFE (estimated with PCM‐B1 and GFDL‐A2 carbon emissions scenarios; IPCC 2007), which may now be conservative estimates (IPCC 2014). These changes are principally due to a combination of sea‐level rise, reduced snowpack and runoff, and continued diversion of freshwater for human uses. Importantly, these factors can greatly fluctuate with extreme events (e.g., such as the ongoing severe drought that has stricken California since 2012) that are also forecasted to increase in magnitude and frequency in the SFE (Cloern et al. 2011). If delta smelt exhibit reduced performance outside the LSZ, these salinity changes may result in reduced habitat for this species under global climate change. Thus, we combined molecular approaches with organismal metrics to: (i) quantify the physiological mechanisms underlying delta smelt's ability to cope with hypo‐ and hyperosmotic stress and (ii) distinguish whether sublethal costs of these physiological changes may contribute to reduced performance in delta smelt outside the LSZ.

We define salinity stress as changes in the saltiness of habitat water that need to be physiologically compensated for to avoid interference with homeostasis and other biological processes (Kültz 2015). We linked transcriptional responses with plasma osmolality, body condition, enzyme activity, and survival to characterize the biological processes involved in achieving homeostasis across a wide range of salinities and considered potential sublethal costs of salinity stress that may limit delta smelt's ability to exploit a broader salinity range *in situ*. We also quantified responses over an exposure time course to capture the rapid as well as downstream signaling and transcriptional regulation that facilitate restoration of osmotic balance and compared these profiles across multiple salinity levels to evaluate thresholds that trigger coordinated molecular responses. Based on the findings of Komoroske et al. (2014), we hypothesized that delta smelt would be able to tolerate and effectively osmoregulate at salinities substantially outside the LSZ conditions, but that achieving this would require large‐scale, coordinated transcriptional and enzymatic responses that could impose sublethal energetic costs on performance. Linking responses across biological levels provides a mechanistic understanding of salinity impacts on this species and provides critical insight into how forecasted salinity changes, particularly under different management scenarios, may affect the physiological performance of estuarine fishes.

## Materials and methods

### Fish culture and holding conditions

Fish were spawned February 2012–2013 and reared at optimal culture temperatures (15.4–16.7°C) determined for delta smelt and 0.2 ppt at the UC Davis Fish Conservation and Culture Laboratory (FCCL; Byron, CA, USA; Lindberg et al. 2013). The delta smelt refuge population breeding program at FCCL incorporates a unique genetic management strategy that includes a variety of methods to minimize inbreeding, maintain genetic representation from the wild population, and maximize genetic diversity (Fisch et al. 2013). We conducted experiments on prespawning adult delta smelt because high‐salinity exposure is most environmentally relevant for this ontogenetic stage (Bennett 2005). Prior to experiments, prespawning adult delta smelt (200–250 days post hatch) were transferred to the UC Davis Center for Aquatic Biology and Aquaculture and held for at least two weeks under a natural photoperiod in 340‐L tanks at 15.5–16.5°C, 2.3 ppt (using artificial sea salt: Instant Ocean, Spectrum Brands, Inc., Blacksburg, VA, USA) based on mean salinity of delta smelt presence in the field (mean salinity = 2.32 ppt, FMWT data 2000–2015; Fig. 1), and fed an *ad libitum* 2:1 mixture of Inve‐NRD commercial feed (Inve Aquaculture, Salt Lake City, UT, USA) and Hikari plankton (Pentair Aquatic Ecosystems, Apopka, FL, USA). Water quality was monitored daily with a YSI 556 water‐quality instrument (YSI Incorporated, Yellow Springs, OH, USA) for pH (8.6 ± 0.38) and dissolved oxygen (90–100% saturation). We used biological filtration, via a custom wet–dry filter that trickled water over Bio‐Balls in an oxygen‐rich chamber, and augmented water quality by exchanging 50% water per week. Ammonia and nitrite were monitored daily using a colorimetric test kit (API, Calfont, PA, USA). All handling, care and experimental procedures used were reviewed and approved by the UC Davis Institutional Animal Care and Use Committee (IACUC Protocol # 16591).

### Salinity exposure experiments

We conducted two acute salinity exposure experiments differing in exposure duration to collect samples for transcriptomics, enzyme activity, osmolality, body condition, and survival. These two sets of experiments served to capture transcriptomic responses that can occur rapidly within minutes to hours (Evans and Somero 2008; Whitehead et al. 2012), as well as the consequent enzymatic changes and aggregate whole organismal alterations that can emerge over timescales of days to weeks (Marshall 2013). We chose to examine gills for transcriptomic and enzyme activity responses because this tissue is a primary interface between the fish's internal and external environment, playing a major role in maintaining homeostasis by transporting ions, oxygen, and water across filament membranes (Evans et al. 2005). We quantified gill Na^+^/K^+^ ATPase activity (NKA) because this enzyme is a key effector for regaining osmotic balance in other fishes (McCormick et al. 2003), and plasma osmolality as a measure of changes in internal solute balance and osmoregulation (Sardella et al. 2004).

#### Experiment 1—Acute exposure and short‐term duration

In the first experiment, we examined effects of acute, short‐term salinity exposures on transcriptomic responses and survival under environmentally relevant salinity treatments (0.4, 2.3, 6.0, 12.0, and 18.0 ppt; Bennett 2005). We transferred 10 randomly selected fish from acclimation tanks into each of the 18.9‐L experimental black round containers (i.e., 5 salinity treatments and 4 time points, with additional replicates for handling controls: 24 containers × 10 fish = 240 fish total; the 2.3‐ppt treatment served as handling control at each time point), fitted with an airstone, drip lines connected to stock water and mesh‐covered drains to maintain 90–100% dissolved oxygen saturation and create flow‐through conditions. We chose this experimental design because delta smelt are schooling, pelagic fish that experience high stress and mortality if cultured, acclimated, or exposed individually or in small groups (Hasenbein et al. 2016), recognizing the trade‐off of possible small tank effects in this component of the study. Containers were placed into a water bath to maintain temperature at 15.4°C (±0.42 SD). After an overnight acclimation mimicking holding conditions (flow‐through water at 2.3 ppt), we checked water quality, fish activity, and subsampled randomly selected fish for ‘pre‐experiment’ baseline transcriptome‐wide assessments. We then increased salinities at constant rates for each treatment over 6 h (approximating a tidal influx time period) to reach target salinities (0.4, 2.3, 6.0, 12.0 and 18.0 ppt). Water was delivered via peristaltic pumps from head tanks with stock salinity solutions using artificial sea salt (Instant Ocean). We recorded water quality hourly during the ramping phase, followed by monitoring at each designated sampling time point (pre‐exposure, 0, 18, and 42 h). At each sampling time point, fish were sacrificed with an overdose of MS‐222 (Tricaine methanesulfonate, Finquel, Argent Chemical Laboratories, Redmond, WA, USA), at a dosage of 50 mg L^−1^ buffered to a neutral pH with sodium bicarbonate (NaHCO_3_), weighed (wet mass ± 0.1 g), and measured (fork length ± 0.5 mm) to assess covariation of fish size and treatments, and gill tissue was immediately dissected and flash frozen by submersion in liquid nitrogen. We chose to focus on gill tissue because in teleost fishes it plays important roles in oxygen uptake, osmotic and ionic regulation, nitrogenous waste excretion, and other critical organismal functions (reviewed in Evans et al. 2005). Samples were stored at −80°C prior to RNA extraction.

#### Experiment 2—Acute exposure and longer‐term duration

In the second experiment, we examined impacts of acute salinity exposures held over longer‐term durations (two weeks) on enzyme activity, osmolality, and survival (see detailed methods in Komoroske et al. 2014). In brief, after an initial acclimation period, fish remained in 340‐L recirculating holding tanks for each salinity treatment (3 replicate tanks × 3 salinities = 9 total). We utilized fish from the same generation as in the acute, short‐term salinity exposures (Experiment 1). Fish from each holding tank were sampled from all replicate and treatment tanks to serve as pre‐experiment baselines, and then salinities were increased following the previously detailed protocol with modified target treatments. Three target salinities were chosen (2.3‐control, 18.5, and 34.0 ppt), based on chronic salinity maximum experiment results (Komoroske et al. 2014), and exposures were conducted after observing low mortality across all treatments in acute Experiment 1. Twenty fish were sampled for each treatment at each designated time point (4–6 fish from each replicate tank): pre‐experiment baseline, 0 h, 6 h, 18 h, 4 day (90 h), 7 day (162 h) and 14 day (330 h) following protocols detailed in the first experiment, except blood was also obtained from the caudal vessel via caudal severance. Blood was collected in microhematocrit tubes and immediately centrifuged at 10 000 ***g*** for five minutes to separate the plasma. This was followed by estimating hematocrit and collection of plasma into 0.5‐mL tubes that were flash frozen and subsequently stored at −80°C until processed. We monitored tanks hourly for mortalities and water‐quality parameters during the gradual salinity increase phase, and at each designated time point and then daily for the three‐week duration of the experiment.

### RNA extraction, amplification, and labeling

Total RNA was extracted from gill tissue using Qiagen RNeasy Kits (Qiagen, Inc., Valencia, CA, USA) according to manufacturer's instructions. RNA concentrations (ng μL^−1^) and purity (A260/A280 and A260/A230 ratios) were determined using a NanoDrop ND1000 Spectrophotometer (NanoDrop Technologies, Inc., Wilmington, DE, USA), and integrity was verified through electrophoresis. Two hundred nanograms of total RNA was then amplified and labeled with Cy3 fluorescent dye using the One‐Color Low Input Quick Amp Labeling kit (Agilent Technologies Inc., Santa Clara, CA, USA) according to the manufacturer's protocol. Briefly, complementary DNA (cDNA) was made from control RNA‐spiked samples followed by complementary RNA (cRNA) synthesis, amplification and Cy3 labeling, and purification. We quantified cRNA concentration and dye incorporation using a NanoDrop ND1000 Spectrophotometer. All samples yielded at least 1.65 μg cRNA and specific activity 6 pmol Cy3 μg cRNA^−1^. To minimize technical artifacts, all reactions were performed simultaneously and individuals from each treatment were randomized in 96‐well plates and subsequently on microarray slides. Dye‐labeled samples were stored in amber tubes at −80°C until microarray hybridization.

### Microarray analysis

We used a delta smelt oligonucleotide microarray (Agilent Technologies Inc.) designed to assess responses to a number of stressors (Jeffries et al. 2015; Komoroske et al. 2015). We performed a total of 72 single‐color microarray hybridizations on 4–6 replicates (gill tissue from individual fish) for the pre‐experiment baseline and salinity challenge × exposure time treatment groups using the custom delta smelt GE microarrays and Agilent Gene Expression Hybridization Kits (Agilent Technologies Inc.). We hybridized amplified cRNA of gill tissue according to Agilent's One‐Color Microarray‐Based Gene Expression Analysis (Low Input Quick Amp Labeling) Protocol. Briefly, prior to hybridization, 1.65 μg of dye‐labeled cRNA sample was combined with 2.2 μL of 25× fragmentation buffer in amber tubes, 11 μL of 10× Gene Expression Blocking Agent and nuclease‐free water to bring the final volume to 55 μL. The fragmentation mix was incubated at 60°C for 30 min, cooled on ice for 1 min, and was stopped by adding 55 μL of 2× Hi‐RPM hybridization buffer. Samples were centrifuged for 1 min, placed on ice, and 100 μL of the mix was loaded onto gasket slides, and the microarray slides were placed on top of the gasket slide. Each slide and gasket slide combination was secured in a single Agilent SureHyb chambers and incubated for 17 h at 65°C, followed by a wash with Gene Expression Wash Buffer 1 at room temperature and Gene Expression Wash Buffer 2 at 37°C according to manufacturer's instructions. All reactions, hybridizations, and washes were completed in the dark. After washing, slides were scanned within 5 h using an Axon GenePix 4000B Scanner and the analysis software GenePix Pro (Molecular Devices, LLC, Sunnyvale, CA, USA). The images were quantified using Feature Extraction v11.5.1.1 (Agilent Technologies Inc.).

We performed normalization, statistical analyses, and graphical representations of microarray data in Genespring (v12.6; Agilent Technologies Inc.) and R (v2.15.2; R‐CoreTeam 2012) and associated packages such as gplot (Warnes et al. 2014). Microarray probes with fluorescent values <100 (approximately 2.5 times the background intensity of an individual array for our dataset), as well as probes that were detected on <50% of the total arrays (suggesting an unreliable probe), were filtered out prior to normalization. Data for the entire array set were quantile normalized and log‐transformed prior to statistical analysis. We assessed expression differences of features between the salinity challenge x exposure time treatment groups using general linear models (GLMs) at *q* ≤ 0.05 (the false discovery rate analog of the corrected *P*‐value). On the microarray, there are two different overlapping probes for each gene sequence that were treated individually in the analyses, validating that duplicate probes representing each gene displayed significant differences among treatments and allowing us to identify the strongest patterns of biological significance in the data. We reported the number of significant probes for each predictor variable or interaction and averaged the values by gene per sample for graphical representation.

For Experiment 1 (acute exposure, short‐term duration), there was no covariation of fish size or weight with salinity treatments or sampling time points, so these metrics were not included in statistical analyses of transcriptomic data. We evaluated multivariate trajectories of gene expression signatures and broad‐scale patterns indicative of physiological responses to salinity over time via nonmetric multidimensional scaling (nMDS; unconstrained ordination) in the R package *vegan* (Oksanen et al. 2013) on probes of primary interest, that is, differential expression for salinity and salinity x exposure time (probes significant at *q* ≤ 0.01 included, averaged by isotig; Bray–Curtis distances, two dimensions, 50 maximum random starts). We also evaluated affected biological processes, which were identified as processes represented by significant genes using the Gene Ontology (GO) categories associated with significant genes using PANTHER (Protein ANnotation THrough Evolutionary Relationship; Mi et al. 2013). Functional analyses on these significant genes were performed using Blast2Go (Conesa et al. 2005). We included gene IDs for probes with *q* ≤ 0.1 for the main effect of challenge salinity and the interaction of challenge salinity × time in analyses and set this gene list against a background list of all annotated genes present on the microarray to evaluate over‐representation of GO categories (analyses automatically removed replicate gene IDs from duplicate probes). Functional groups were considered significantly enriched using Fisher's exact tests at FDR ≤ 0.05. Secondly, we individually confirmed and identified the molecular functions and biological processes of the genes of primary interest via http://uniprot.org and supporting literature. The latter served to gain a more in‐depth understanding of the underlying components of the broad biological process categories identified by functional analyses.

Finally, we evaluated transcriptional changes in relation to key genes and physiological responses to salinity stress previously identified in the literature (Evans and Somero 2008; Whitehead et al. 2012) to distinguish similarities and differences between delta smelt and other euryhaline fishes. To do this, we used UniProt's *Function* description as well as *GO molecular function* and *GO biological process* information to assign each gene of primary interest (i.e., using a conservative approach including genes with *q* ≤ 0.01 for salinity or salinity x exposure time, duplicate probes for each gene averaged) to one or more principal categories of (i) osmosensing and signaling, (ii) ion and cell volume regulation, (iii) cellular transport and phosphorylation, (iv) cell proliferation and normal cellular cycle processes, and (v) metabolism and respiration. During assignment, we also considered creation of novel categories to identify unique physiological mechanisms employed by delta smelt. However, we did not find robust evidence (i.e., congruent significant patterns across multiple genes) to provide strong enough support for the inclusion of novel classifications in our results. We conducted *a posteriori* analyses on assigned genes using Tukey's honestly significant difference tests (corrected *P* ≤ 0.05) based on significance of main effects or their interaction in main GLMs for each gene. We focus reporting of these results on significant differences between handling controls and overall salinity treatments (i.e., main effect of salinity ≤0.05, and salinity × time point interaction ≥0.05), or salinity treatments at specific time points, when appropriate (i.e., salinity × time point interaction ≤0.05).

### Organismal, osmolality, body condition and enzyme activity measures

During both experiments, we monitored survival hourly during the salinity increase phase and at each designated sampling time point, as well as daily in Experiment 2 (acute exposure, longer‐term duration). In Experiment 2, length and weight measurements were also used to calculate a body condition index (BCI; defined as weight/length^3^) as an indicator of overall physiological state (Bolger and Connolly 1989) and compared among salinity treatments over time. We quantified organismal‐level effects of salinity and exposure time via (i) BCI using a linear mixed model (LMM; replicate tank as a random effect) in R packages lme4 and lmerTest (Bates et al. 2015; Kuznetsova et al. 2015), as well as metrics described in Komoroske et al. (2014), (ii) survival using a generalized linear mixed model (GLMM), and (iii) salinity tolerance via a GLM.

Due to the small size of delta smelt, only 2–10 μL of plasma was obtained per individual. To avoid confounding issues of pooling samples, a small sample holder (AC‐063) was used in conjunction with a vapor pressure osmometer (Vapro 5600; Wescor Inc., Logan, UT, USA) to analyze and quantify plasma osmolality. When possible, 2.5‐μL plasma samples were processed in duplicate to assess consistency, and replicates were averaged prior to analysis. Total plasma osmolality is expressed as mmol kg^−1^ (referring to kg of sample). The activity of gill Na^+^/K^+^‐ATPase activity (NKA) was measured utilizing McCormick's (1993) microplate method, adapted for small fish (i.e., whole gill used due to small tissue sizes). Whole gill was homogenized in 500 μL of homogenizing buffer (250 mm sucrose, 10 mm Na_2_ EDTA, 50 mm imidiazole, 0.5% Na deoxycholic acid) and centrifuged for one minute at 5000 ***g*** at 4°C (Eppendorf, Hamburg, Germany). To determine NKA, 10 μL of homogenate was loaded onto a 96‐well microplate and 200 μL of assay solution (in the presence or absence of ouabain) was added to each well. A kinetic reading (340 nm for 10 min at 25°C) was performed (Synergy HT microplate reader; Biotek, Winooski, VT, USA), and NKA activities were determined as the ouabain‐inhibited fraction of total ATP hydrolysis and the conversion of NADH to NAD^+^. Activities were standardized by measurement of total protein (bicinchoninic acid; BCA Protein Assay Kit; Pierce, Rockford, IL, USA) according methods described by Smith et al. (1985), and NKA activities were expressed as μmol of ADP x mg protein^−1^ × h^−1^. We applied LLMs to assess effects of salinity and exposure time on plasma osmolality and NKA activity, including replicate tank as a random effect.

## Results

### Salinity effects on organismal, osmolality, body condition, and enzyme activity measures

**Figure 2:**
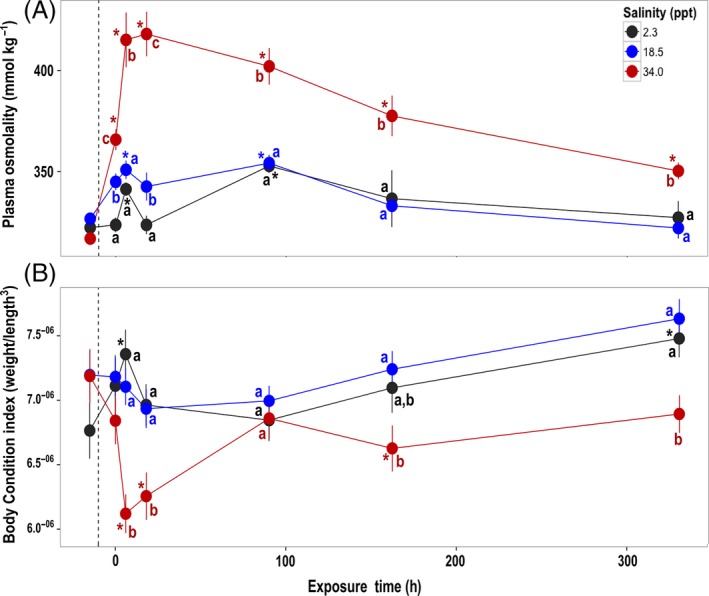
Effects of exposure time and salinity in Experiment 2 (acute exposure, longer‐term duration) on delta smelt (A) blood plasma osmolality (mmol kg^−1^) and (B) condition index = weight (g)/fork length (mm)^3^; mean ± SEM for each salinity per exposure time point. Fish were taken for pre‐experiment samples randomly from each tank under control conditions (2.3 ppt) prior to 6 h gradual increases to target salinities (0‐h time point denotes when tanks reached target salinity); tanks remained at target salinities (2.3 control, 18.5, or 34.0 ppt) for the duration of the experiment. Asterisks indicate where responses within a treatment are significantly different from the pre‐experiment value, while lettering designates differences between treatments within each time point.

In Experiment 1 (acute exposure, short‐term duration: gradual salinity increases to targets of 0.4, 2.3, 6, 12, and 18 ppt), mortality was only observed at 0.4 ppt (0, 10, and 10% at 0, 18, and 42 h, respectively) and 18 ppt (0, 0, and 10% at 0, 18, and 42 h, respectively). In Experiment 2 (acute exposure, longer‐term duration), significant mortality was observed only in the highest salinity treatment (34.0 ppt), with mortality occurring principally between 18 and 90 h (survival = 81.5% at 90 h; Komoroske et al. 2014). Plasma osmolality was significantly affected by salinity, exposure time, and their interaction (Table 1; Fig. 2A). Osmolality was most strongly affected in the 34.0‐ppt treatment, rapidly increasing followed by decreasing after 90 h back toward control levels. After 330 h, fish exposed to 34.0 ppt still had higher osmolality relative to the 18.5 and 2.3 ppt groups; however, it was greatly below peak levels at 6 and 18 h. Fish exposed to 18.5 ppt had increased plasma osmolality at initial time points, but returned to control levels within 90 h. Delta smelt body condition index (BCI) was also affected, specifically with fish exposed to 34.0 ppt exhibiting lower body condition after two weeks of exposure (Table 1; Fig. 2B). In contrast, Na^+^/K^+^‐ATPase activity (NKA) was highly variable across salinity and exposure times and did not show any significant differences between treatments (Figure S1).

**Table 1:** Effects of salinity and exposure time in Experiment 2 (acute exposure, longer‐term duration) on delta smelt (A) blood plasma osmolality (mmol kg^−1^) and (B) body condition index = weight (g)/fork length (mm)^3^

	df	SS	MS	*F*	*P*‐value
(A) Osmolality					
Salinity	2/207	89 184	44 592	92.615	<0.0001
Exposure time (h)	6/207	64 642	10 774	22.376	<0.0001
Salinity × Exposure	12/207	44 296	3691	7.667	<0.0001
(B) Body condition index					
Salinity	2/6.31	0.141	0.070	11.79	0.007
Exposure time (h)	6/400	0.131	0.022	3.665	0.001
Salinity × Exposure	12/399	0.177	0.015	2.467	0.004

### Transcription signatures and functional analyses

**Figure 3:**
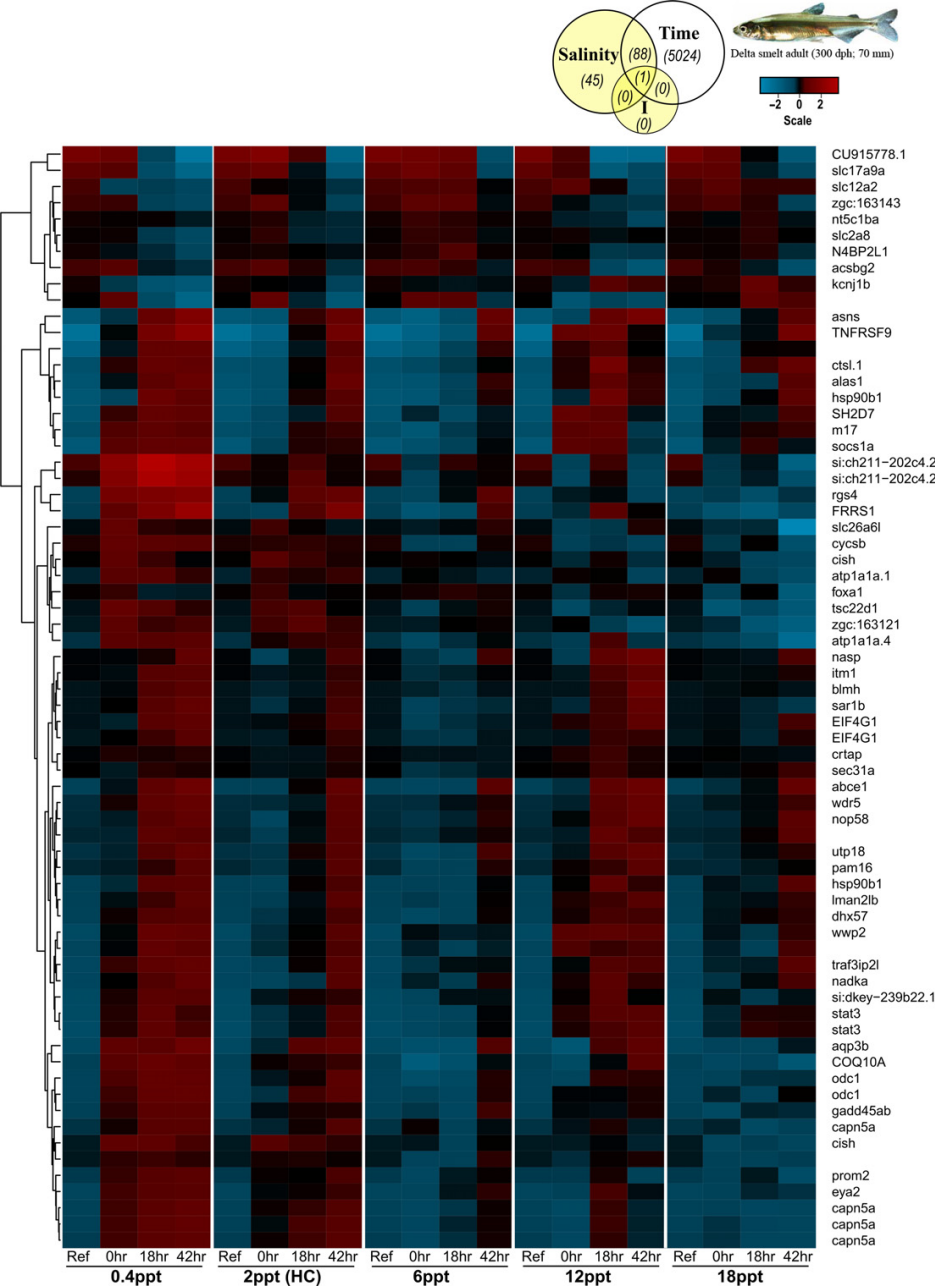
Heat map of microarray genes in Experiment 1 (acute exposure, short‐term duration) with *q* ≤ 0.01 for main effect of salinity (salinity) and the interaction of salinity x exposure time (I) (yellow shading, Venn diagram). Duplicate probes and replicates were averaged across treatments for visualization, while numbers in the Venn diagram refer to total numbers of significant probes for each factor or interaction; *n* = 4–6 individual fish for each salinity and exposure time group. Genes clustered on averaged probe similarity (dendrogram displayed on left, based on Euclidean distance matrix and complete agglomeration); a three‐color scale (value bar depicted above) was applied to visualize fold changes of mean normalized values between treatments (See Supplementary information for expanded heat map and table for all genes *q* ≤ 0.05).

**Figure 4:**
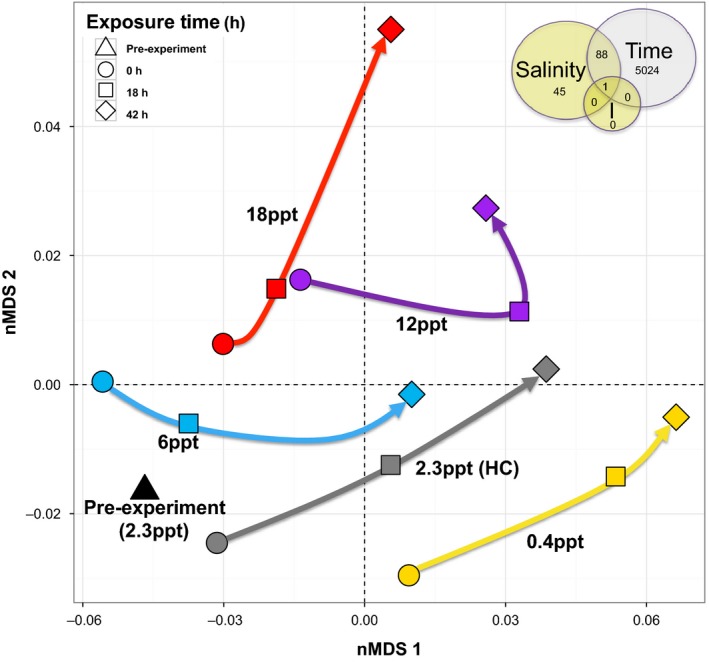
Two‐dimensional nonmetric multidimensional scaling plot of microarray genes in Experiment 1 (acute exposure, short‐term duration) with *q* ≤ 0.01 for main effect of salinity (salinity) and the interaction of salinity x exposure time (I) (yellow shading, Venn diagram). Shapes correspond to exposure time (triangle = pre‐experiment, circle = 0 h, i.e. when target salinities were reached after 6 h gradual increases or decreases, square = 18 h, diamond = 42 h) and color corresponds to salinity treatment (black = 2.3 ppt pre‐experiment, yellow = 0.4 ppt, gray = 2.3 ppt handling control, blue = 6.0 ppt, purple = 12.0 ppt, red = 18.0 ppt). Arrows overplotted to visualize mean trajectory of transcriptomic changes over the exposure time course of the experiment.

In Experiment 1, of a total of 17 596 probes on the delta smelt microarray, 622 and 8615 were differentially expressed for salinity and time main effects, respectively, as well as 87 for their interaction at *q* ≤ 0.05 (Figure S2 and Table S1). Of the genes affected by salinity, the majority were also affected by time (Fig. 3, depicting genes *q* ≤ 0.01), underscoring the importance of time course in evaluation of gene expression responses to environmental stress. For transcription signatures, two convergent nMDS solutions were found for two dimensions after five iterations (stress = 0.0825) and overall mean transcriptome trajectories of lower salinity treatments (0.4 and 6 ppt) were more similar to that of the handling control relative to higher treatments (Fig. 4). However, transcription signatures at 0 h (i.e., when fish reached target salinities after the 6 h of gradual increase phase) were substantially offset from the pre‐experiment position for all treatment groups, reflecting rapid gene expression responses to changes in salinity conditions.

**Figure 5:**
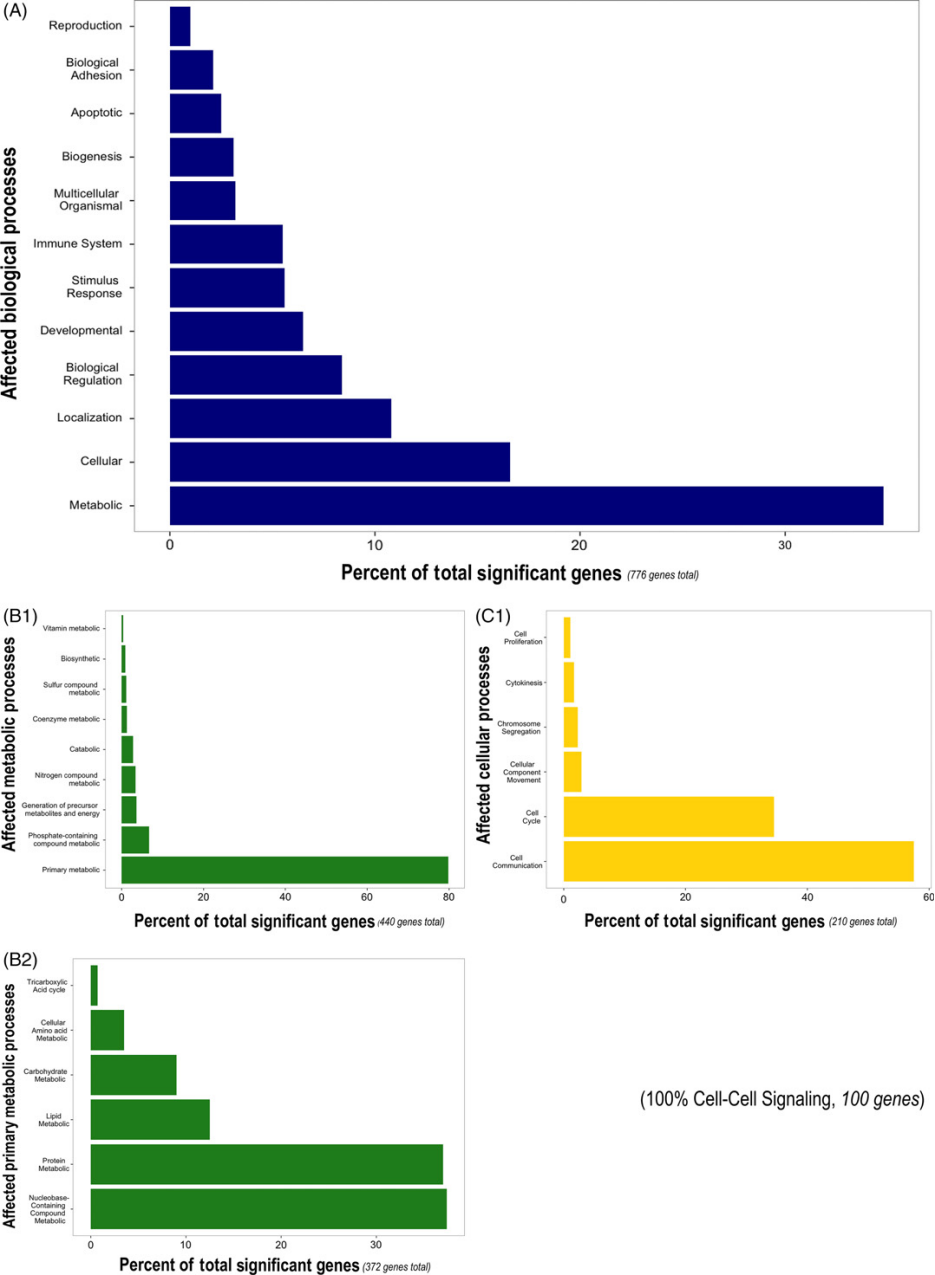
Composite bar charts for biological processes represented by genes in Experiment 1 (acute exposure, short‐term duration) affected by probes of primary interest (combined list of genes displaying differential expression at *q* ≤ 0.1 for salinity main effect and salinity x exposure time interaction). Panels represent: (A) overall biological processes, (B1) subcategories of metabolic processes in (A), (B2) subcategories of primary metabolic processes in (B1), (C1) subcategories of cellular processes in (A).

Of the total 1653 probes selected for functional analyses (*q* < 0.1 for salinity or salinity × time), we were able to assign 1497 ENSEMBL IDs; of those, PANTHER mapped 768 to zebrafish genome annotated genes, while 163 were unmapped (duplicate probes automatically removed). Functional analyses revealed that cellular and metabolic processes were the two main biological processes represented in genes significantly affected by salinity over the experimental time course (Fig. 5). Within the metabolic processes group, the majority of genes represented primary metabolic processes, including lipid, protein, and carbohydrate metabolism. The cellular processes group largely consisted of genes involved in cell cycle and cellular communication, with cell–cell signaling genes making up the entire latter group. Statistically over‐represented biological processes included multiple signaling pathways (e.g., chemokine receptor binding, cytokine‐mediated signaling pathways, and JAK‐STAT signaling cascade regulation) as well as potassium ion transport (Table S2).

### Transcriptional responses to salinity stress

#### Osmosensors and signaling

**Figure 6:**
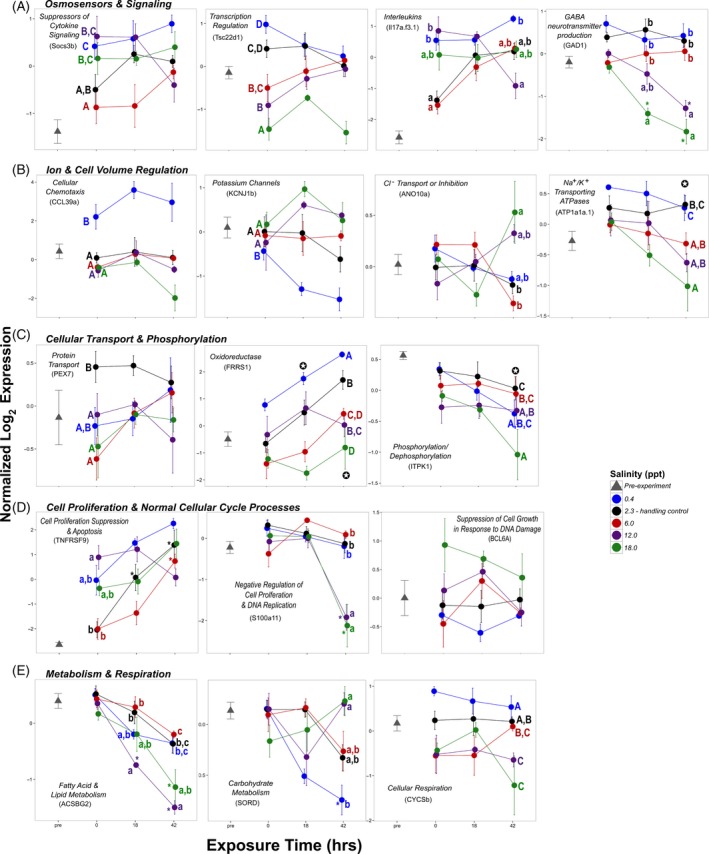
Effects of salinity and exposure time on the expression of genes in Experiment 1 (acute exposure, short‐term duration) representative of key biological processes (mean ± SEM for each salinity per exposure time point). Y‐axes units are normalized log2 expression, reversed for ease of interpretation of transcriptional changes (i.e., lower number is higher transcription). Post hoc analyses of biological interest were conducted where appropriate as determined by main models (see Materials and methods). Points and error bars represent mean ±SEM; colored symbols and letters correspond to treatment colors in the legend. Uppercase letters denote main effect contrasts for salinity treatments, while lowercase letters denote pairwise contrasts between salinities within each time point. Asterisks denote statistical difference from time point 0; ✪ denotes main effect contrasts, while * denotes pairwise contrasts.

A large number of genes involved in numerous signaling pathways were altered by exposure to different salinities. Interestingly, despite the small absolute change, fish exposed to a decrease from 2.3 ppt to 0.4 ppt rapidly upregulated multiple genes involved in the regulation of G‐protein signal transduction and pathways (regulator of G‐protein signaling 4, RGS4, and platelet‐activating factor receptor, PTAFR). Interleukin‐17A/F‐3 (IL17a/f3), which regulates cytokine activity and cell surface receptor signaling pathways, and the suppressor of cytokine signaling 3b (SOCS3b) involved in JAK‐STAT signaling cascades were also upregulated (Figs 6A and S2; Table S1). G‐protein and cytokine signaling were also altered in fish exposed to salinity increases to 12 and 18 ppt, but many responses differed in trajectory (e.g., downregulation of RGS4) or manifested via different particular genes such as neuropeptide B (NPB) cytokine‐inducible SH2‐containing protein (CISH), G‐protein‐coupled receptor 81 (HCAR1‐1), and suppressor of cytokine signaling 2 (SOCS2). Expression of the transcriptional repressor TSC22 domain family protein 1 (Tsc22d1) and glutamate decarboxylase (Gad1), which catalyzes GABA neurotransmitter production, also significantly decreased in fish exposed to these hyperosmotic stress treatments (Figs 6A and S2; Table S1).

#### Ion and cell volume regulation

Transcriptional responses indicated that delta smelt made physiological adjustments at the molecular level to regain ion and cell volume homeostasis. Hyperosmotic conditions affected the expression of several genes involved in cellular adhesion, regulating ion and water passage across cell junctions such as claudin (CLDNA), tensin‐1 (TNS1), and collagen alpha‐2 chain (COL6A2; Figure S2 and Table S1). In contrast, chemokine CCL (CCL39a), which promotes cellular chemotaxis, was strongly upregulated at 0.4 ppt (Fig. 6B). Delta smelt exposed to 0, 6, and 12 ppt also downregulated frizzled‐9 (FZD9a), a key gene involved in Rho‐GTPase activity that has been found in other osmotic stress studies to play important roles triggering signaling cascades that induce morphological changes (Di Ciano‐Oliveira et al. 2006).

The Na^+^/K^+^/2 Cl^−^ cotransporter (SLC12A2), which plays a critical role in ionic balance and cell volume regulation by mediating Na^+^ and Cl^−^ reabsorption, and the potassium inwardly rectifying channel isoform b (KCNJ1b) were strongly downregulated at 0.4 ppt (Figs 6B and S2; Table S1). In contrast, at 18‐ppt transcription increased for SLC12A2, and decreased for solute carrier protein family 26 member 6 (SLC26A6), inward rectifier potassium channel 16 (KCNJ16), aquaporin‐3 (AQP3b; a membrane protein that can function as a channel to facilitate water transport across cell membranes) and prominin‐2 (PROM2), which negatively regulates pinocytosis and can increase protein phosphorylation. Additionally, multiple genes involved in Na^+^/K^+^ ATPase mechanisms were downregulated at 18 ppt, including Na^+^/K^+^‐transporting ATPase subunit *α*‐1 (ATP1A1A.4, ATP1A1A.1), Na^+^/K^+^‐transporting ATPase subunit *β* (ATP1B1, regulates the number of sodium pumps transported to the plasma membrane via assembly of *α*‐*β* heterodimers), and Na^+^/K^+^‐transporting ATPase subunit *γ* (ATP1G1; Figs 6B and S2; Table S1). Finally, at both 12 and 18 ppt, anoctamin‐10 (ANO10a), involved in the transport or inhibition of anion (i.e., Cl^−^) conductance (Milenkovic et al. 2010), and protein nipal2 (NIPAL2; a magnesium ion transmembrane transporter) were up‐ and downregulated, respectively, at 42 h.

### Cellular transport and phosphorylation

In addition to genes specifically involved in ion transport across cell membranes, the transport of proteins and other substances within and across cell membranes was also affected by increased salinity (Figs 6C and S2; Table S1). Peroxisomal targeting signal 2 receptor (PEX7), which plays an essential role in peroxisomal protein import, was downregulated at 6, 12 and 18 ppt, while ferric‐chelate reductase (FRRS1) was strongly upregulated at 0.4 ppt, suggesting that iron transport from the endosome to the cytoplasm may play a role in delta smelt's hyposmotic stress response. Multiple genes that promote dephosphorylation of existing effector proteins also displayed lowered expression in response to hyperosmotic stress, including inositol‐tetrakisphosphate 1‐kinase (ITPK1; lower at 12 and 18 ppt), thiamine triphosphatase (THTPA; lower at 12 and 18 ppt), protein phosphatase 1 regulatory subunit 14 (PPP1R14ba; lower at 12 ppt at 42 h), and protein phosphatase 1 regulatory subunit (PPP1R15B; lower at 18 ppt). Interestingly, THTPA was also downregulated at 0 ppt, suggesting that this gene may be involved in modulating phosphorylation states of effector proteins under both hypo‐ and hyperosmotic stress responses.

#### Cell cycle regulation and cellular stress

Fish exposed to 12 and 18 ppt exhibited altered expression of multiple genes controlling cell cycle, chromosomal division, and DNA replication. Most significant changes occurred at 42 h, including the downregulation of protein S100‐A11 (S100a11) and the upregulation of centromere protein U (CENPU), protein zwilch (ZWILCH), DNA primase small subunit (PRIM1), and lymphocyte‐specific helicase (HELLS). However, tumor necrosis factor receptor superfamily member 9 (TNFRSF9), a gene involved in apoptosis and the suppression of cell proliferation, and genes involved in reactive oxygen species (ROS) production and oxidative stress responses (NADPH oxidase activator 1, NOXA1, and hypoxia upregulated protein 1, HYOU) were upregulated in fish exposed to 12 ppt at 0 h. Additionally, at 18 ppt, the expression increased for B‐cell lymphoma 6 protein (BCL6A), a transcriptional repressor that can negatively regulate cell growth in the face of genotoxic stress (i.e., in response to DNA damage stimulus), while the transcription factor hepatocyte nuclear factor 3‐alpha (FOXA1), involved in positive regulation of mitosis and glucose homeostasis, was strongly downregulated at 42 h. Interestingly, two genes indicative of DNA damage and apoptosis displayed divergent patterns among hyper‐ and hyposmotic stress: eyes absent homolog 2 (EYA2) and growth arrest and DNA damage‐inducible protein GADD45 (GADD45) were up‐ and downregulated at 0.4 and 18 ppt, respectively.

#### Metabolism and respiration

Genes indicative of changes in fatty acid and lipid metabolism were significantly altered in both hypo‐ and hyperosmotic stress treatments relative to handling controls, including acetyl‐coenzyme A dehydrogenase (ACADM; downregulated at 0.4, 12 and 18 ppt), long‐chain fatty acid‐CoA ligase (ACSBG2; downregulated at 12 ppt at 18 and 42 h; Fig. 6E), and prostaglandin E synthase 2 (PTGESL; upregulated at 12 and 18 ppt). Delta smelt also upregulated ornithine decarboxylase 1 (ODC1) particularly in the hyposmotic treatment; ODC1 is the rate‐limiting enzyme in polyamine synthesis, and has been found to be an important component of hyposmotic stress response in other species (Lockwood and Somero 2011; Whitehead et al. 2012). Additionally, genes involved in carbohydrate metabolism, cellular glucose homeostasis and glycogen synthesis or breakdown were modulated, such as mannose‐6‐phosphate isomerase (MPI; downregulated at 18 ppt at 42 h), protein phosphatase 1 regulatory subunit 3C‐B (PPP1R3CB; downregulated at 12 ppt at 0 h), and sorbitol dehydrogenase (SORD; upregulated at 12 and 18 ppt, and downregulated at 0.4 ppt). Cellular respiration genes involved in the mitochondrial respiratory chain (cytochrome c‐somatic B, CYCS‐b, and coenzyme Q‐binding protein, COQ10A) were also downregulated in hyperosmotic treatments.

## Discussion

Our data demonstrate that delta smelt have the capacity for coordinated molecular responses to effectively osmoregulate and regain homeostasis across a broad range of salinities. By employing large‐scale transcriptomic changes, fish rapidly adjusted to considerable increases in osmotic gradients, as well as to the reversal from hypo‐ to hyperosmotic conditions. These abilities are particularly evidenced by the regulation of internal osmolality to control levels in fish exposed to 18.5 ppt after just six hours. However, at the highest salinity (34.0 ppt), fish displayed reduced body condition even with unlimited food resources, and functional analyses identified that lipid, protein, and carbohydrate metabolism played major roles in delta smelt's compensatory responses to salinity stress outside the low‐salinity zone (LSZ) conditions. These findings align with theoretical models and empirical evidence in other species showing that osmoregulatory processes are energetically expensive (Morgan and Iwama 1991; Boeuf and Payan 2001; Kültz 2015), and that such environmental stress can impose sublethal costs due to the additional energy needed to recover and maintain homeostasis (Calow and Forbes 1998; Sokolova et al. 2012). Although delta smelt are physiologically euryhaline (i.e., are able to tolerate 0.4 – 34.0 ppt), the cumulative costs associated with physiological adjustments required to achieve homeostasis across a large, fluctuating salinity gradient may be higher than the continual maintenance cost for homeostasis within LSZ salinities. The evolution of such a homeostatic set point that differs from isosmotic conditions has been observed in other fishes (Papakostas et al. 2012) and could be reinforced by factors not directly related to osmoregulation, but that covary with salinity and offer fitness benefits (i.e., food availability or predator avoidance). This combination could further constrain the abilities of delta smelt to effectively exploit habitat outside the LSZ, corresponding with strong *in situ* correlations of this species with both environmental and ecological parameters (Fig. 1; Bennett 2005; Feyrer et al. 2007). Thus, forecasted mean salinity increases of 2.2–4.5 ppt in the San Francisco Estuary (SFE) are not likely to induce mortality, but these environmental changes will probably further constrict habitat that provides optimal conditions for performance and reproductive output in delta smelt. Identifying the physiological mechanisms that organisms use to cope with an individual stressor, and how those responses may impose sublethal costs, is an important step toward understanding of how multiple global change factors influence species’ fitness and, ultimately, persistence.

A large number of genes involved in many molecular pathways were altered by salinity over the exposure time course in delta smelt, supporting our hypothesis and previous work demonstrating that regaining homeostasis in the face of osmotic stress requires complex and coordinated physiological responses beginning at the transcriptional level (Evans and Somero 2008; Whitehead et al. 2012). Transcriptional signatures and their trajectories over time were more similar among 0.4, 2.3 (handling controls) and 6 ppt, relative to 12 and particularly 18 ppt. These patterns align with the concept that changes in the magnitude of osmotic gradients require physiological adjustments within an organism's hyposmotic regulatory strategy, while the reversal of the osmotic gradient to hyperosmotic conditions entails switching physiological strategies that potentially utilize different underlying mechanisms. Interestingly, some genes responded similarly in delta smelt under both hypo‐ and hyperosmotic challenges, indicating they perhaps play common roles in both responses, while others clearly differentially responded to the divergent conditions.

Fish exposed to salinity challenges outside LSZ conditions (i.e., 0.4‐below LSZ, 12 and 18 ppt above LSZ) exhibited the greatest transcriptional changes. Many of the responsive genes were associated with similar physiological mechanisms identified in other estuarine fishes (i.e., cell signaling, re‐establishment of ionic and osmotic balance, suppression of normal cell cycle regulation, and altered metabolic processes) and were in accordance with shifting resource allocation underlying euryhalinity (reviewed in Evans 2010). Many of the specific gene identities involved in these processes were similar to other euryhaline fishes examined to date. This is perhaps suggestive of the use of some conserved as well as convergent molecular pathways in euryhalinity (Kültz 2015), but the current availability of well annotated genomes for only a few fish species in functional genomic databases limits robust comparative analyses. However, recent advances in teleost genomic resources (Rondeau et al. 2014) hold exciting promise to facilitate future studies comparing these mechanisms across closely related and divergent euryhaline species.

Body condition and survival of delta smelt exposed to 18.5 ppt were not significantly affected, and blood plasma osmolality returned to control levels after 6 h. These results reveal that rapid physiological adjustments allowed fish to quickly regain homeostasis at this salinity, which was surprising given the well‐known sensitivity of delta smelt to environmental stress (Bennett 2005; Hasenbein et al. 2013; Komoroske et al. 2015). Yet both the body condition and survival of fish exposed to 34.0 ppt were significantly reduced, indicating that the costs of coping with strong hyperosmotic stress negatively impacts fitness. Importantly, we observed these negative impacts under experimental conditions with unlimited food resources. The ability of delta smelt to rapidly regain homeostasis at 18.5 ppt may have been dependent on access to ample energy reserves, which may not always be the case *in situ*. It has been proposed that a driving force of selection in the evolution of euryhalinity is access to energy‐rich estuarine environments (Kültz 2015). For estuarine fishes like delta smelt, inhabiting such fluctuating salinity environments is likely a perpetual balancing act between adequately dispensing enough resources to prevent osmotic stress‐related damage and compromise cellular function, without unnecessarily diverting cellular resources away from growth and reproduction (Evans 2010). However, there is increasing evidence of resource limitation in SFE ecosystems due to both natural variation and food web shifts induced by invasive species (Cloern and Jassby 2012). Thus, if delta smelt face food limitation *in situ*, it is very possible that the energetic costs of mounting osmoregulatory responses may negatively impact performance and survival at more moderate levels of hyperosmotic stress. Additionally, effects may be more pronounced for other delta smelt life stages such as early juveniles that require relatively high energetic investment for rapid growth. Further research investigating the dynamics of osmoregulatory responses and food limitation would provide critical insight into the effects of the interactions of these important factors.

Contrary to our hypothesis, we did not observe changes in enzymatic activity associated with osmotic stress; Na^+^/K^+^‐ATPase activity (NKA) was not affected by salinity or exposure time. This was surprising, particularly in light of clear evidence of regulation of internal osmolality in delta smelt. In teleosts, a hallmark effector of osmoregulation in the gills is the modulation of NKA along with related cotransporters to effectively maintain osmotic and ionic balance (Evans et al. 2005; Hwang and Lin 2013). Studies in a variety of fishes have documented increased NKA activity with osmotic stress (Marshall 2013) and NKA ‘isoform switching’, in which one isoform is upregulated at low salinities while another is downregulated and vice versa at increased salinities (Richards et al. 2003; Bystriansky et al. 2006; Urbina et al. 2013). Additionally, delta smelt downregulated gene expression of multiple NKA subunits at high salinities, but we did not detect any reciprocal increases in the transcription of alternate isoforms. Several Na^+^/K^+^/2 Cl^−^ cotransporters (NKCC's) and other solute carrier proteins and inward rectifier potassium channels displayed opposing transcriptional patterns at low versus at high salinities. Expressional changes and osmoregulatory function of these cellular components in gills, and particularly ionocytes, have been found in other studies in both freshwater and estuarine fishes (Marshall 2003; Wood 2011; Dymowska et al. 2012). Taken together, these data suggest that delta smelt may be able to effectively regain homeostasis at increased salinities without the upregulation of NKA transcription or activity, but perhaps via adjustments of other pathway components or mechanisms (Evans and Somero 2008). As an estuarine fish, delta smelt may have evolved alternate strategies to cope with rapid salinity fluctuations, potentially employing physiological mechanisms that offer faster responses to changing conditions (e.g., phosphorylation, maintaining higher constitutive protein levels; Kültz 2013). We did observe altered gene expression of kinases and phosphatases that are involved in post‐translational modification of proteins through phosphorylation and dephosphorylation to heighten or repress their activities, respectively. Phosphorylation of NKCCs in response to salinity stress has been observed in other species of euryhaline fishes (Flatman 2002; Flemmer et al. 2010), as well as other cellular components involved in both osmosensing signaling pathways and effector proteins (Evans and Somero 2008; Kültz 2015). These rapid mechanisms may be especially important for osmoregulators inhabiting estuarine environments such as delta smelt. As the application of high‐throughput molecular studies across a greater diversity of fishes continues to expand, it will provide further insight into the evolutionary and ecological contexts under which species employ differential physiological strategies such as cellular remodeling, transcriptional, and post‐translational mechanisms to cope with salinity stress.

Due to anthropogenic freshwater diversion in the SFE that results in higher salinity habitats, management emphasis is frequently focused on effects of hyperosmotic stress. However, our data also highlight the large‐scale transcriptomic changes delta smelt initiated to cope with freshwater osmoregulatory challenges, such as those endured during upstream spawning migration to freshwater. Despite the small absolute salinity change from LSZ conditions, freshwater reduces ion availability to very low levels, and freshwater fishes typically have high‐affinity ion‐uptake pumps to effectively cope with these environmental conditions. However, estuarine fishes do not necessarily possess these adaptations, and may need to make other physiological adjustments to maintain homeostasis (Marshall 2013). This is supported by delta smelt's upregulation of genes for enzymes and cotransporters related to solute transport at 0.4 ppt, as well as those involved in preventing pinocytosis. Similar transcriptional responses as well as intolerance to freshwater acclimation have been observed in killifish species that typically otherwise exhibit euryhaline capabilities (Whitehead et al. 2011; Patterson et al. 2012). While the specific costs of physiological adjustments to cope with freshwater in delta smelt are not yet clear, spawning is an energetically demanding activity in the absence of additional costs of migration or coping with environmental stress, re‐emphasizing the need for adequate energetic resources for successful reproduction and persistence in this largely annual species.

Identifying underlying physiological mechanisms and costs of environmental stress can help identify how and when we might expect organisms to suffer sublethal impacts or increased susceptibility to other stressors (Helmuth et al. 2005). In addition to behavioral and ecological studies quantifying biotic interactions, this information may be especially important when governing entities have an assortment of management ‘levers’ they can adjust to affect conservation or restoration efforts (e.g., reservoir releases or reducing water diversions to regulate river flows). For example, if the absence of delta smelt in high‐salinity waters was due solely to their inability to tolerate these conditions, a natural management focus would be to maintain suitable habitat within the tolerable salinity range. However, given that delta smelt actually possess the physiological ‘machinery’ to cope with conditions outside this range, but cofactors such as energetic costs may play critical roles limiting their performance, it may be beneficial to also focus efforts on other ecological factors (e.g., food resource supply and community structure). Shifts in phytoplankton and zooplankton communities in conjunction with increasing invasive species richness and abundance has greatly altered food web dynamics in the SFE (Cloern and Jassby 2012), emphasizing the importance of ecological interactions in understanding global change impacts on SFE native species (Haller et al. 2014; Lee et al. 2015). While inclusion of multiple stressors and ecological complexities is challenging, the absence of considering them may lead to ineffective management actions (e.g., population decline despite maintenance of the LSZ zone, due to inadequate food supply or adaptation of marine predators allowing them to expand into LSZ waters). Our work isolating the physiological mechanisms underpinning delta smelt's shorter‐term salinity stress responses lays the foundation for future studies investigating multiple stressors such as environmental stress and resource limitation, as well as longer‐term exposure effects.

## Conflict of interest

The authors declare no conflict of interest.

## Data archiving statement

Microarray data have been deposited in the Gene Expression Omnibus (http://www.ncbi.nlm.nih. gov/geo/) with the Accession no. GSE72772.

## Supporting information


**Figure S1.** Effects of exposure time and salinity in Experiment 2 (acute exposure, longer‐term duration) on delta smelt gill Na^+^/K^+^ ATPase enzyme activity (mean ± SEM for each salinity per exposure time point).

**Figure S2.** Heat map of microarray genes in Experiment 1 (acute exposure, short‐term duration) with *q* ≤ 0.05 for main effect of salinity and the interaction of salinity x exposure time.

**Table S1.** Genes represented by microarray probes of primary interest (differential expressionat *q* < 0.05 for salinity challenge main effect and salinity challenge x recovery time interaction).
**Table S2.** Over‐represented biological processes of genes significantly affected by main effect of salinity or salinity x exposure time interaction as determined by Blast2Go functional analyses (note: no biological processes were significantly under‐represented).
