# Supplementary figures and images for: Sublethal salinity stress contributes to habitat limitation in an endangered estuarine fish
Source: Evol Appl. 2016 Jun 8;9(8):963–81. doi: 10.1111/eva.12385 (PMC4999527; doi:10.1111/eva.12385)

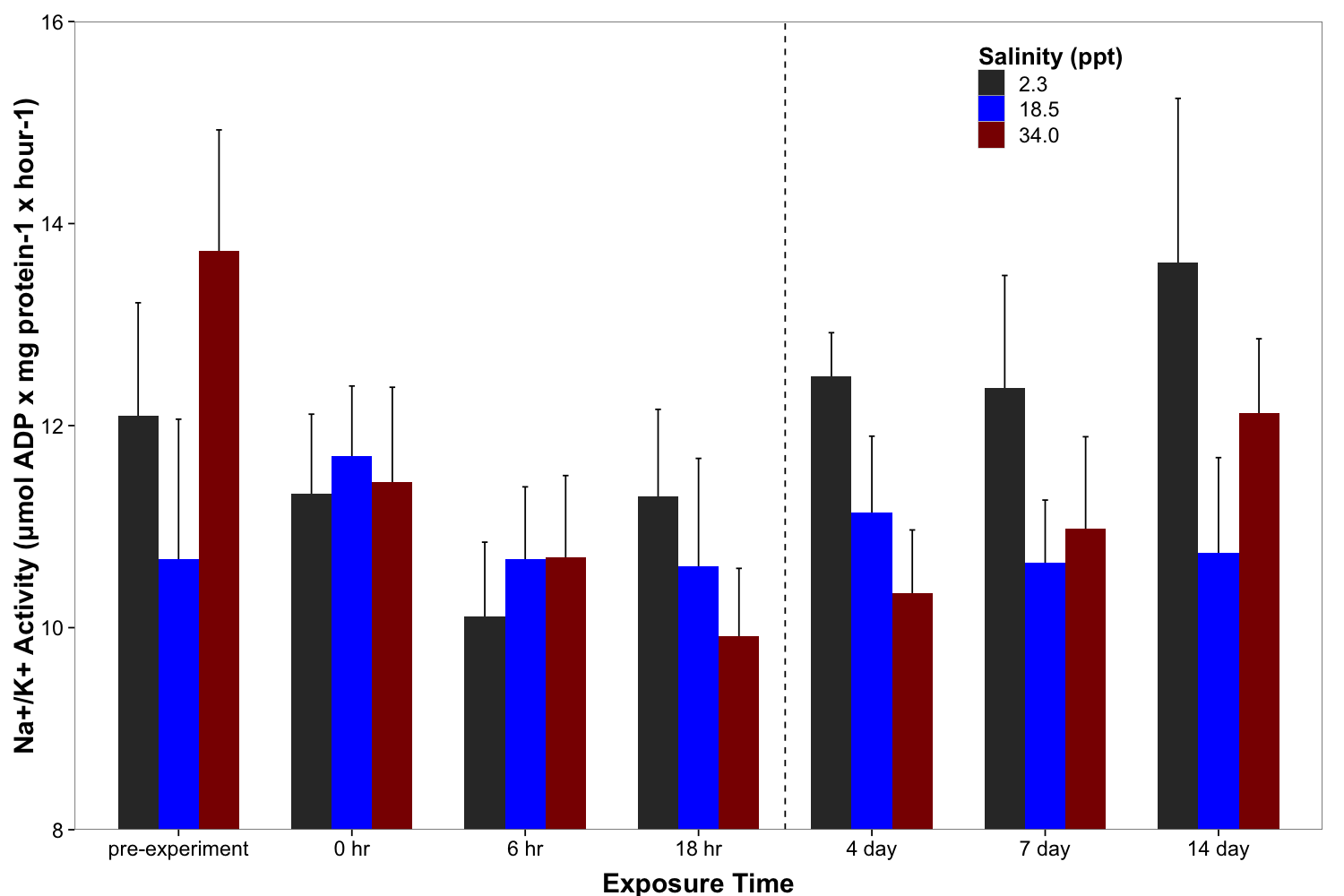

Supplement: Supplementary file 1 — Figure S1. Effects of exposure time and salinity in Experiment 2 (acute exposure, longer‐term duration) on delta smelt gill Na+/K+ ATPase enzyme activity (mean ± SEM for each salinity per exposure time point). [file EVA-9-0963-s001.png]

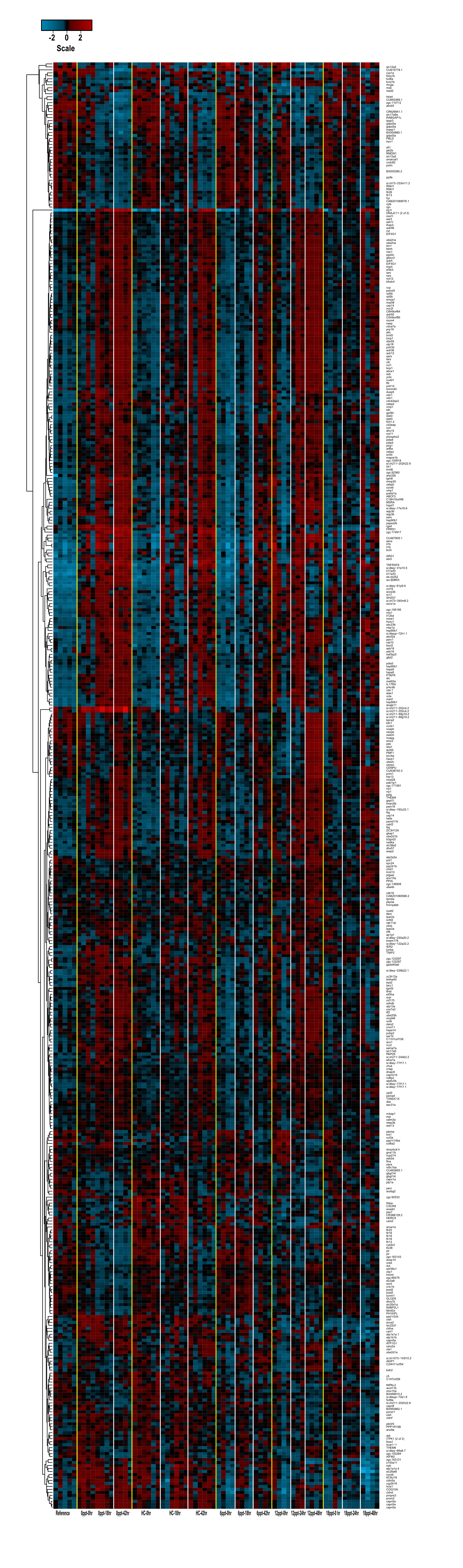

Supplement: Supplementary file 2 — Figure S2. Heat map of microarray genes in Experiment 1 (acute exposure, short‐term duration) with q ≤ 0.05 for main effect of salinity and the interaction of salinity x exposure time. [file EVA-9-0963-s002.png]
